# Age‐Related Low Frequency Amplitude Differences in Resting‐State Blood Oxygenation Level‐Dependent Signal in the Cerebellum

**DOI:** 10.1002/hbm.70541

**Published:** 2026-05-10

**Authors:** Jessica A. Korte, Christopher J. Steele, Wilsaan M. Joiner, Audrey P. Fan

**Affiliations:** ^1^ Department of Biomedical Engineering University of California, Davis Davis California USA; ^2^ Department of Psychology Concordia University Montreal Quebec Canada; ^3^ School of Health, Concordia University Montreal Quebec Canada; ^4^ Department of Neurology Max Planck Institute of Human Cognitive and Brain Sciences Leipzig Germany; ^5^ Department of Neurology University of California, Davis Davis California USA; ^6^ Department of Neurobiology, Physiology and Behavior University of California, Davis Davis California USA

**Keywords:** aging, cerebellum, human connectome project, low frequency fluctuations, resting‐state fMRI

## Abstract

There is a growing interest to study cerebellar contributions to aging outside of traditional sensory processing and motor tasks. While cerebellar aging analyses typically utilize functional connectivity (FC) to study functional differences with age, this study aimed to identify a marker of healthy aging based on resting state blood oxygenation level dependent (BOLD) signal dynamics in the cerebellum. To do this, we investigated both Amplitude of Low Frequency Fluctuations (ALFF) and fractional ALFF (fALFF), semi‐quantitative metrics of the strength of the BOLD signal. We found that fALFF is a highly repeatable metric of cerebellar function that demonstrates a significant increase in BOLD signal fluctuations at 0.008–0.1 Hz in cerebellar regions Crus I and II with aging. Furthermore, cerebellar fALFF of these regions was associated with FC to cortical regions across separate scanning sessions. These results highlight age‐related differences in spontaneous cerebellar dynamics, particularly in regions tied to the frontal cortex, motivating the use of fALFF as a potential biomarker of healthy aging and motivate the need to incorporate the cerebellum in existing models of brain network changes with age.

## Key Points

- Fractional amplitude of low frequency fluctuations (fALFF) in the cerebellum (Crus I/II) demonstrates significant age‐related differences between young adults and older adults.
- fALFF is a highly robust metric within the cerebellum, demonstrating high repeatability across separate sessions.
- Increased fALFF is associated with increased connectivity to frontal regions within the default mode network.

## Graphical abstract

This study found that fractional amplitude of low frequency fluctuations in cerebellar Crus I/II is significantly greater in older adults as compared to younger adults. The higher amplitude in this region is associated with increased connectivity to cortical regions, and may be characteristic of healthy aging.

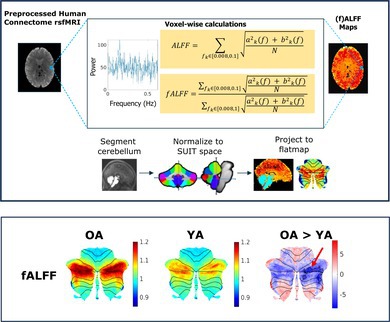

## 1 Introduction

In typical aging, a number of behavioral processes are maintained, such as implicit (unconscious) sensorimotor adaptation (Huang et al. 2017; Reuter et al. 2018; Vachon et al. 2020), and predictive timing (Filip et al. 2019; Johari et al. 2018), all of which are supported by cerebellar circuitry (King et al. 2023; Nettekoven et al. 2024; Stoodley 2012; Stoodley and Schmahmann 2009). Yet the cerebellum undergoes large neuronal loss with age, particularly of Purkinje and granule cells (Andersen et al. 2003; Hogan et al. 2011; Walløe et al. 2014). The concept of cerebellar reserve, the ability of the cerebellum to compensate for pathological changes, highlights the cerebellum's potential to support neural processes in aging and disease (Mitoma et al. 2021, 2023). However, a majority of functional cognitive aging studies do not include the cerebellum in their analysis (MacDonald and Pike 2021). As a result, there has been growing interest to understand the effect of aging specifically on cerebellar function (Arleo et al. 2024; Bernard 2022) and how these changes contribute to broader circuitry variations.

Resting‐state functional magnetic resonance imaging (rsfMRI) is a neuroimaging technique commonly used to study the functional dynamics of elderly cohorts as it is non‐invasive, not confounded by task demands (i.e., task differences that may reflect physical aging as opposed to cognitive aging) and can be used to study the functional dynamics that support neural function in vivo. While most rsfMRI studies measure blood oxygenation level dependent (BOLD) signal changes using functional connectivity (FC; (Biswal et al. 1995; Biswal et al. 2010; Smitha et al. 2017)), voxel‐wise measures such as the amplitude of low‐frequency fluctuations (ALFF; (Yu‐Feng et al. 2007)) and fractional‐ALFF (fALFF) capture the intensity of BOLD signal fluctuations between 0.008–0.1 Hz, reflecting spontaneous neural oscillations. These measures exhibit high reproducibility (Cahart et al. 2023; Golestani et al. 2017; Zuo et al. 2010) and demonstrate characteristic differences across various brain disorders (Hrybouski et al. 2025; Zhang et al. 2019), suggesting they contain neuronal and physiological information that differ in clinical populations. Aging‐related cortical changes have also been identified using ALFF and similar metrics, with older adults producing lower amplitude of BOLD within 0.01–0.1 Hz (Yang et al. 2018; Zhong and Chen 2022). Cerebellar aging has primarily been studied through cortico‐cerebellar FC (Bernard et al. 2013, 2020; Gellersen et al. 2021; Hausman et al. 2020; de Uwisengeyimana et al. 2020). To our knowledge, no study has explicitly focused on aging‐related changes of (f)ALFF in the cerebellum.

Prior work has reported positive associations between ALFF and FC, raising the possibility that regions with greater low‐frequency BOLD amplitude may also exhibit stronger functional coupling (Di, Kim, et al. 2013; Mascali et al. 2015; Tommasin et al. 2017). However, neurovascular reactivity, the ability of cerebral blood vessels to dilate in response to a stimulus, can also influence both BOLD amplitude and inter‐regional correlations, providing a physiological mechanism by which (f)ALFF and FC may covary independently of neuronal coupling (Liu et al. 2017). In the context of aging, older participants demonstrate decreased BOLD amplitude and variability (Garrett et al. 2010, 2013; Millar et al. 2020) as well as decreased long‐range FC (Zhou et al. 2023). Aging can also reduce the hemodynamic response through decreased neurovascular coupling (Tarantini et al. 2017; Yabluchanskiy et al. 2021), which in turn can reduce the BOLD amplitude and SNR, resulting in lower FC (Liu et al. 2017). Age‐related changes in cerebellar‐cortical connectivity appear to differ across cerebellar sub‐lobular regions, with some studies reporting increased FC to the cortex in older adults (Mccarthy et al. 2014; Tomasi and Volkow 2012), potentially reflecting regional preservation, compensatory recruitment, or changes in neurovascular dynamics. Together, these findings indicate that local BOLD amplitude differences in the cerebellum may be indicative of age driven shifts in its connectivity to cortical regions.

The goal of this study was to identify a robust metric for studying functional differences within the cerebellum associated with age. To compare differences between young adults and older adults, we utilized data from a public cohort of rsfMRI data via the Human Connectome Project (HCP) Young Adult S1200 and Aging datasets. Our first step consisted of a control analysis. In both cohorts, we evaluated and compared potential physiological/data quality confounds with our metrics of interest, ALFF and fALFF, within the cerebellum. After characterizing these differences, we calculated regional ALFF and fALFF within the cerebellum to identify aging‐related BOLD frequency differences within the cerebellum, accounting for the potential confounds identified in our control analysis. Next, we conducted a repeatability and reliability assessment to establish the reliability of age group differences across scan sessions. We then explored aging‐related differences in functional connectivity between key cerebellar regions identified using resting‐state frequency‐based metrics and the cortex.

## 2 Materials and Methods

### 2.1 Participants

The HCP Young Adult S1200 has an available database of 860 participants acquired on a Siemens 3T Connectom scanner at Washington University (Marcus et al. 2011) while HCP‐Aging has 726 participants acquired on Siemens 3T Prisma across four different sites (Harms et al. 2018), 338 of whom are 60 and older. An a priori power analysis was conducted to determine the required sample size using a two‐tailed independent samples *t*‐test with target power of 0.8 and a significance level of 0.05. Required sample sizes were estimated across a range of effect sizes (Cohen's *d*). A minimum of 100 participants (50 per group) was required to detect group‐level differences at a reasonable effect size (*d* = approximately 0.5). To accommodate potential exclusions during quality control, we randomly selected data from 70 young adults (YA; 35 female) and 70 older adults (OA; 35 female) from the HCP Young Adult S1200 and Aging datasets. All YA were between 18 and 35 years old, while OA were between 65 and 79. Participants were considered if they successfully completed two resting‐state scanning sessions on two separate days for a total of four rsfMRI scans and HCP documentation indicated no quality issues during scan acquisition. Quality control included: visual inspection to ensure adequate rsfMRI coverage of the cerebellum, removal of participants with large amounts of movement (described below), and hand‐correcting cerebellum white/gray matter segmentation. After quality control, 61 YA and 59 OA subjects remained.

### 2.2 Imaging Data

Details on the acquisitions for HCP‐YA 1200 can be found in (Glasser et al. 2016; Van Essen et al. 2012). Briefly, each participant's T1‐weighted MPRAGE scan (0.7 mm isotropic), T2‐weighted FLAIR scan (0.7 mm isotropic), 1 baseline resting state (rs) scan (TR: 720 ms, phase‐encoding: R‐L, spatial resolution: 2 mm isotropic, duration: 14.4 min, multi‐band factor: 8) and one follow‐up rs‐scan separated by at least 1 day were used for analysis. YA rsfMRI data was truncated to the first 541 samples (a duration of approximately 6.5 min) to achieve a scan duration comparable to OA acquisition.

**FIGURE 1:**
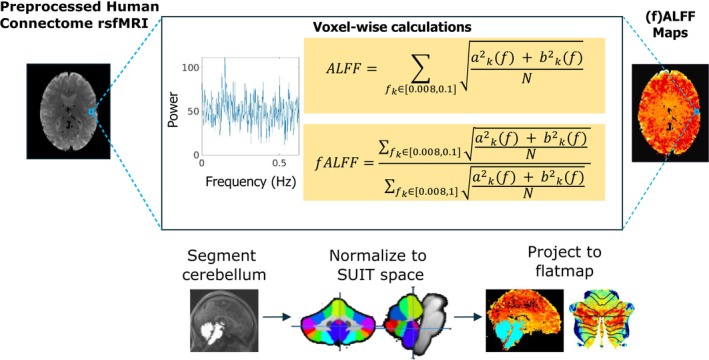
Analysis of ALFF and fALFF measures (A) Schematic for the voxel‐wise calculation of the Amplitude of Low Frequency Fluctuations (ALFF) and fractional ALFF (fALFF). (B) Processing steps for isolating the cerebellum using the Spatially Unbiased Infra‐tentorial Template (SUIT) atlas.

For OA, the T1‐weighted MPRAGE (0.8 mm isotropic), T2‐weighted FLAIR (0.8 mm isotropic), and two separate rsfMRI acquisitions on two separate days (TR: 800 ms, phase‐encoding: P‐A, spatial resolution: 2 mm isotropic, duration: 6.5 min, multi‐band factor: 8) for a total of four rsfMRI images were used for analysis (Figure 1B). Further details on the acquisitions for HCP‐A can be found in (Bookheimer et al. 2019).

### 2.3 Image Preprocessing

Distortion effects were corrected using spin echo scans acquired on the same day, processed with FSL's topup. Preprocessing of the distortion‐corrected rsfMRI followed the minimal preprocessing pipeline (Glasser et al. 2013), which includes: correction for head movement and spatial distortion, registration to structural data, rigid alignment to the MNI152 atlas, and functional artifact removal. No smoothing was performed. The first five timepoints were discarded to account for initial relaxation effects. CONN (RRID:SCR_009550) release 20.a (Nieto‐Castanon and Whitfield‐Gabrieli 2020) was used for preprocessing. Frame outliers were identified in the Artifact Detection Tools (ART) toolbox within CONN and were defined by movement greater than ±0.5 mm or global BOLD signal changes above three SD. Subjects that had greater than 10% frame outliers in an individual session were removed from analysis. Framewise displacement (FD) was computed using CONN. This was performed in subject space at each timepoint by considering a 140 × 180 × 115 mm bounding box around the brain and estimating the largest displacement among six control points placed at the center of these bounding‐box faces (Nieto‐Castanon 2020).

Denoising was conducted in CONN and did not include detrending, as previous methodological studies have found that detrending can bias (f)ALFF metrics and decrease repeatability (Woletz et al. 2019). Six principal components were included as confound regressors, derived from: linear regression of movement, CSF, and white matter (Woletz et al. 2019). The denoised signal was then Fourier transformed and bandpass filtered to [0.008–0.1] Hz. Voxel‐wise maps of ALFF were calculated as the average power in the frequency filtered spectrum (Figure 1A). fALFF was similarly calculated by dividing the average power in the [0.008–0.1] Hz range by the power across the full, non‐filtered spectrum. (f)ALFF maps were normalized to the average whole brain (f)ALFF to enable comparison across participants (Küblböck et al. 2014; Xi et al. 2012).

### 2.4 Statistical Analyses

After (f)ALFF normalization, cerebellar segmentation, and removal of movement outliers, SPM12 (RRID:SCR_007037) (Penny et al. 2006) release 12.7771 was used to register structural and functional maps to the Spatially Unbiased Infra‐tentorial Template (SUIT) cerebellum lobular template (Diedrichsen 2006; Diedrichsen et al. 2009) (Figure 1B). This atlas and normalization method was developed for more accurate spatially normalized functional analyses. The resolution of the scans is sufficient to reflect volumetric results at sub‐lobular levels (the average volume of each lobule being approximately 114 cm^3^ (Diedrichsen 2006; Zheng et al. 2023)), and similar resolution has been used at this level of analysis—for example, (King et al. 2019). SPM12 was used for all group‐level statistical comparisons. Statistical results were projected onto SUIT flatmaps to enable visualization (Diedrichsen and Zotow 2015). This flatmap reflects the average over several folia for each lobule and is used only for visualization of results.

### 2.5 Control Analysis

The BOLD signal is aliased with physiological effects such as heart rate and respiration [0.05–0.15 Hz] (Attarpour et al. 2021; Lee et al. 2023) due to low sampling rates (Liu et al. 2017; Power et al. 2017). Compared to cortical regions, BOLD signal in the cerebellum exhibits similar heart rate‐related fluctuations but is more sensitive to respiration (van der Zwaag et al. 2015), with inferior regions being particularly vulnerable to these physiological confounds. Aging has been found to increase cardiac effects in the BOLD signal of white matter regions (Viessmann et al. 2019); moreover, cortical BOLD amplitude is highly associated with cardiovascular and cerebrovascular factors (Tsvetanov, Henson, Jones, et al. 2021). All of this indicates that the cerebellar BOLD signal is susceptible to respiratory and cardiac effects, which are differentially affected by aging and will affect resulting (f)ALFF values. Accordingly, we wanted to evaluate how physiology may explain group‐level differences in cerebellar ALFF/fALFF measures. To do this, global BOLD (gBOLD), the average BOLD signal across the whole brain, was correlated with cerebellar BOLD at a voxel level. gBOLD signal is reflective of a multitude of factors such as vascular coupling, scanner noise, head movement, and even potentially neuronally‐driven slow oscillations, but is often attributed to “physiological noise” (Addeh, Williams, et al. 2025; Keilholz et al. 2017). Physiological noise typically references systemic effects from heart rate (Chang et al. 2009) and respiration (Liu et al. 2017; Raitamaa et al. 2021), two factors highly affected by age. gBOLD was used as a proxy of physiology as opposed to direct measurements from HCP data due to the low number of participants with usable recordings (Fan et al. 2025). Results were evaluated using a lenient threshold (uncorrected *p* < 0.001, cluster size > 10) (Bernard et al. 2013; Kwak et al. 2010). Additionally, differences in gray matter volume probability (GMVp) maps were compared between groups to identify areas where age‐related structural degradation may contribute to frequency differences. Finally, the temporal signal‐to‐noise (tSNR) ratio was calculated for each participant's scan to address potential BOLD amplitude differences between cohorts. This is a voxel‐wise metric calculated after preprocessing the data, and is the mean BOLD signal divided by the standard deviation over the scan duration. Again, a lenient threshold was used to evaluate group‐level statistics.

### 2.6 Analysis

ALFF and fALFF ((f)ALFF) maps were compared between younger versus older groups to identify cerebellar regions that are sensitive to age using a voxel‐wise one‐way *t*‐test. All statistical analyses were evaluated with a family‐wise error (FWE) height correction of p‐FWE < 0.05. Only clusters with a minimum voxel size of 10 voxels are shown graphically and considered for FC analysis. Mean framewise displacement as reported by CONN, cerebellar gray matter probability volume, and correlation with global BOLD were used as covariates for group comparisons.

### 2.7 Repeatability and Reliability Assessment

Repeatability refers to the variation of repeat measurements made on the same participant, while reliability refers to the inherent variability between participants (Bartlett and Frost 2008). To quantify regional variability in ALFF/fALFF, each participant's cerebellar map was averaged within each SUIT anatomical ROI, excluding the dentate regions due to resolution constraints, for a total of 28 individual regions. For each ROI, we obtained one mean ALFF/fALFF value per participant for session 1 and session 2. Test–retest reliability was then assessed by computing an Intraclass Correlation Coefficient (ICC) value (Shrout and Fleiss 1979) for each cerebellar ROI across participants, using the paired regional values from the two resting‐state sessions. Additionally, Bland–Altman plots for (f)ALFF over the whole cerebellum were generated to compare repeatability of both metrics across separate sessions.

### 2.8 Functional Connectivity Analysis

To explore how local cerebellar BOLD dynamics relate to broader functional coupling, the largest cluster from fALFF group differences was selected as a seed region for functional connectivity (FC) analysis in CONN (region indicated by the red arrow in Figure 3). Each individual's time series within this cerebellar ROI was extracted and used for voxel‐wise correlation to the cortex. The resulting Fisher's correlation map was then normalized to the MNI152 atlas and smoothed with a 6‐mm full‐width at half‐maximum (FWHM) Gaussian kernel. This process was repeated for each participant's separate resting‐state session.

For each individual, the average connectivity between each cortical region defined by the Yeo/Buckner atlas (Buckner et al. 2011) and the cerebellar fALFF ROI was computed for each session. These values were then concatenated across sessions, resulting in FC of 104 separate regions with repeated observations per subject. A multivariate linear mixed effects model was generated with FC as the dependent variable and mean framewise displacement (mFD), sex, session, and group assignment (younger versus older, with Younger Adults as the reference group) in MATLAB, with participant as a random effect to account for two sessions per person. To control for multiple comparisons, a positive False Discovery Rate of p‐FDR < 0.001 (Benjamini and Yekutieli 2005) was applied to the resulting correlations. ROIs that demonstrated significant group‐level differences were reported.

## 3 Results

### 3.1 Control Analysis

**FIGURE 2:**
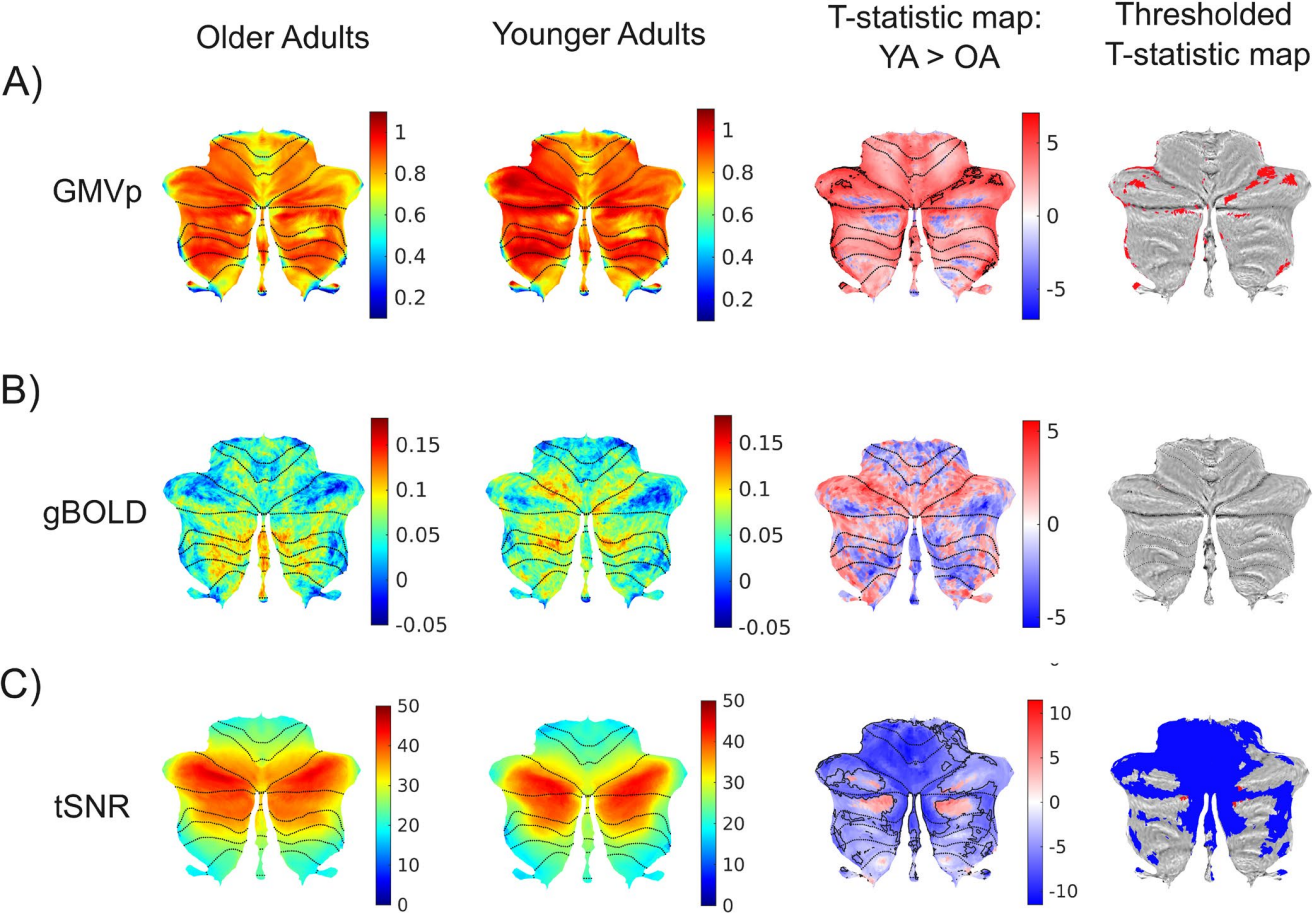
Comparison of global BOLD (gBOLD) correlation, cerebellar gray matter probability, and temporal signal‐to‐noise (tSNR) between age groups. Group‐averaged results for respective control analyses for Older Adults (OA; left column), Young Adults (YA; middle column), and the resulting t‐statistic map (red indicates where YA > OA, blue indicates where OA > YA). The thresholded t‐statistic results are shown in the right‐most column. Areas where YA > OA are shown in red, while OA > YA is shown in blue. (A) Group‐level gray matter volume probability (GMVp) map comparisons. Areas that passed multiple comparison correction are outlined in black. The largest of these clusters is 16 voxels, suggesting minor differences between the two groups. (B) Resulting beta values of the correlation of gBOLD signal with voxel‐wise cerebellar BOLD between OA and YA. There were no voxels that survived the lenient threshold (*p* < 0.001, p‐FWE < 0.05, cluster size > 10 voxels). (C) Group‐level temporal signal to noise ratio (tSNR) map comparisons. Overall, OA had greater tSNR than YA. Regions that survived the lenient threshold (*p* < 0.001, p‐FWE < 0.05, cluster size > 10 voxels) are outlined in black in the t‐statistic column and highlighted in the thresholded column.

OA had significantly higher mFD than YA for session 1 (OA = 0.141 ± 0.063, YA = 0.087 ± 0.026, *p* < 0.0001) and session 2 (OA = 0.142 ± 0.056, YA = 0.093 ± 0.026, *p* < 0.0001). GMVp was significantly higher in YA than OA in medial regions of Crus I/II (Figure 2A); however, correcting for GMVp in regions of ALFF and fALFF differences did not alter cluster extent of group differences. Correlation of gBOLD with voxel‐wise cerebellar BOLD was not significantly different between groups (Figure 2B). When the resulting group T‐statistic map was visually compared with a venous probability map, there was high visual correspondence with venous locations (Figure S1). To quantify similarity to group‐level ALFF and fALFF differences, the resulting t‐statistic map from gBOLD correlation analysis was binarized using an uncorrected threshold level of *p* < 0.05. Binarized clusters from group‐level ALFF and fALFF analysis were compared using a dice coefficient. ALFF t‐statistic maps had a dice value of 0.404, while fALFF t‐statistic maps had a dice value of 0.0196. tSNR comparison revealed that overall, OA had higher signal than YA (Figure 2).

### 3.2 Aging‐Related Differences in Cerebellar BOLD Low‐Frequency Amplitude

**FIGURE 3:**
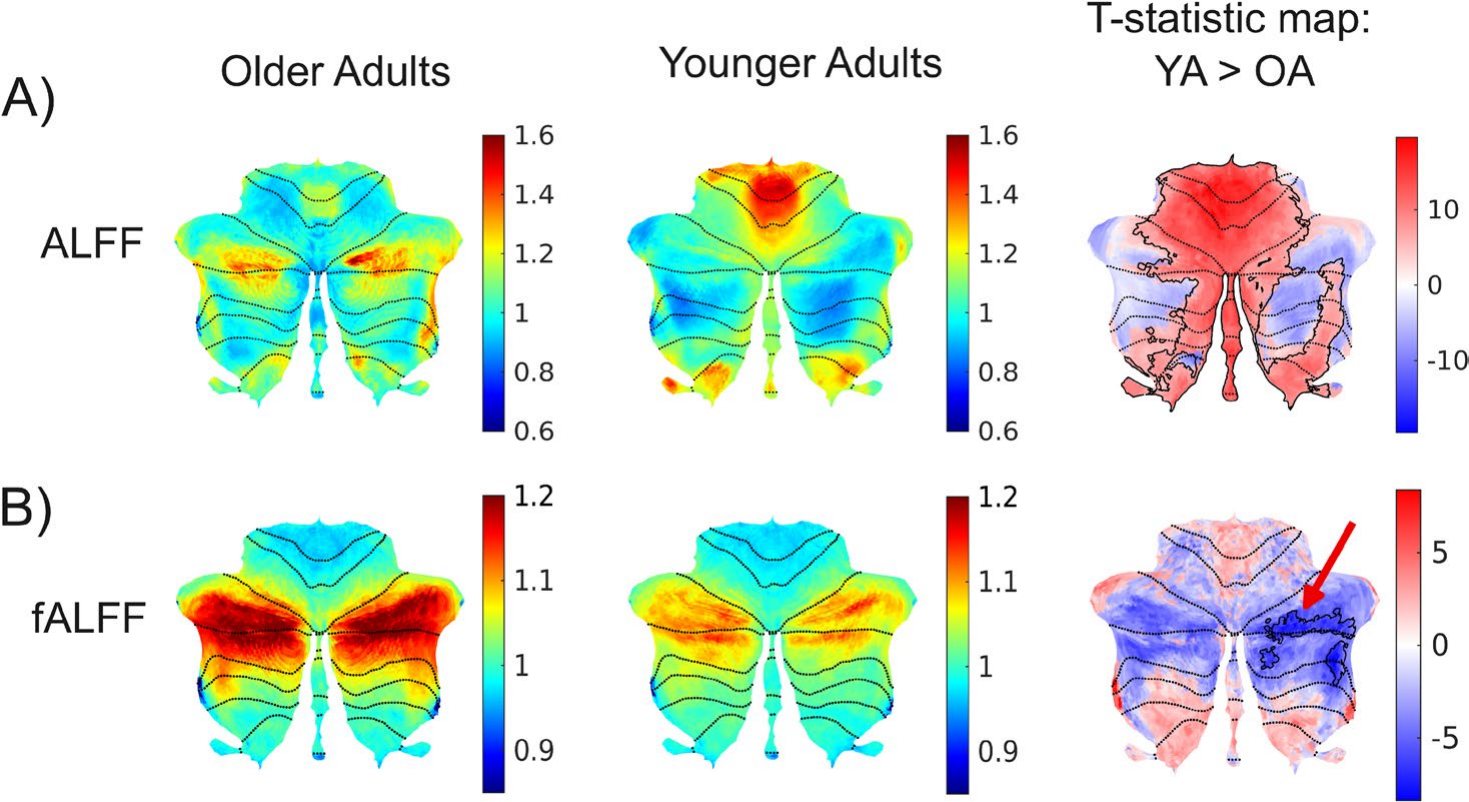
Age‐related differences in regional cerebellar ALFF and fALFF values. Group‐averaged ALFF maps for Older Adults (OA; left column), Young Adults (YA; middle column), and the resulting t‐statistic map after accounting for covariates (p‐uncorrected < 0.001, p‐FWE < 0.05, minimum extent of 10 voxels; right column). Red values indicate areas where YA had higher ALFF than OA, while blue indicates the reverse. Regions that survive cluster correction are outlined in black on the t‐statistic map. (A) Regions that demonstrate significant group differences for ALFF values are located medially in Lobules I‐V, and laterally in Lobules VIIIB, IX and X. In these clusters, YA had higher ALFF (Mean = 1.920, SD = 0.829, 95% CI: [1.712, 2.128]) than OA (Mean = 1.527, SD = 0.613, 95% CI: [1.371, 1.683]). (B) Regions that demonstrate significant group differences in fALFF values are located laterally in Crus I, Crus II and Lobule VIIB. In these clusters, OA had higher fALFF (Mean = 1.675, SD = 0.888, 95% CI: [1.448, 1.902]) than YA (Mean = 1.543, SD = 0.816, 95% CI: [1.338, 1.748]). The red arrow indicates the Crus I/II cluster used for functional connectivity analysis.

ALFF revealed significant differences between OA and YA within the cerebellum after cluster correction. Specifically, YA clusters exhibited significantly higher ALFF than OA in bilateral Lobules I–V, VIIIB, IX, and X (p‐uncorrected < 0.001, p‐FWE < 0.05, cluster size > 10; Figure 3A). Cohen's effect size of group differences (*d* = 0.54) suggests a moderate practical significance. No significant clusters remained when the *t*‐test was conducted in the opposite direction.

OA had higher fALFF in Crus I and II across both hemispheres as compared to YA after multiple comparison correction (p‐uncorrected < 0.001, p‐FWE < 0.05, cluster size > 10; Figure 3B). Cohen's effect size value (*d* = 0.16) suggests that this group difference is more difficult to detect. No significant clusters remained when the *t*‐test was conducted in the opposite direction. The largest cluster in Crus I/II was used for subsequent functional connectivity analysis.

### 3.3 Repeatability and Reliability Assessment

**FIGURE 4:**
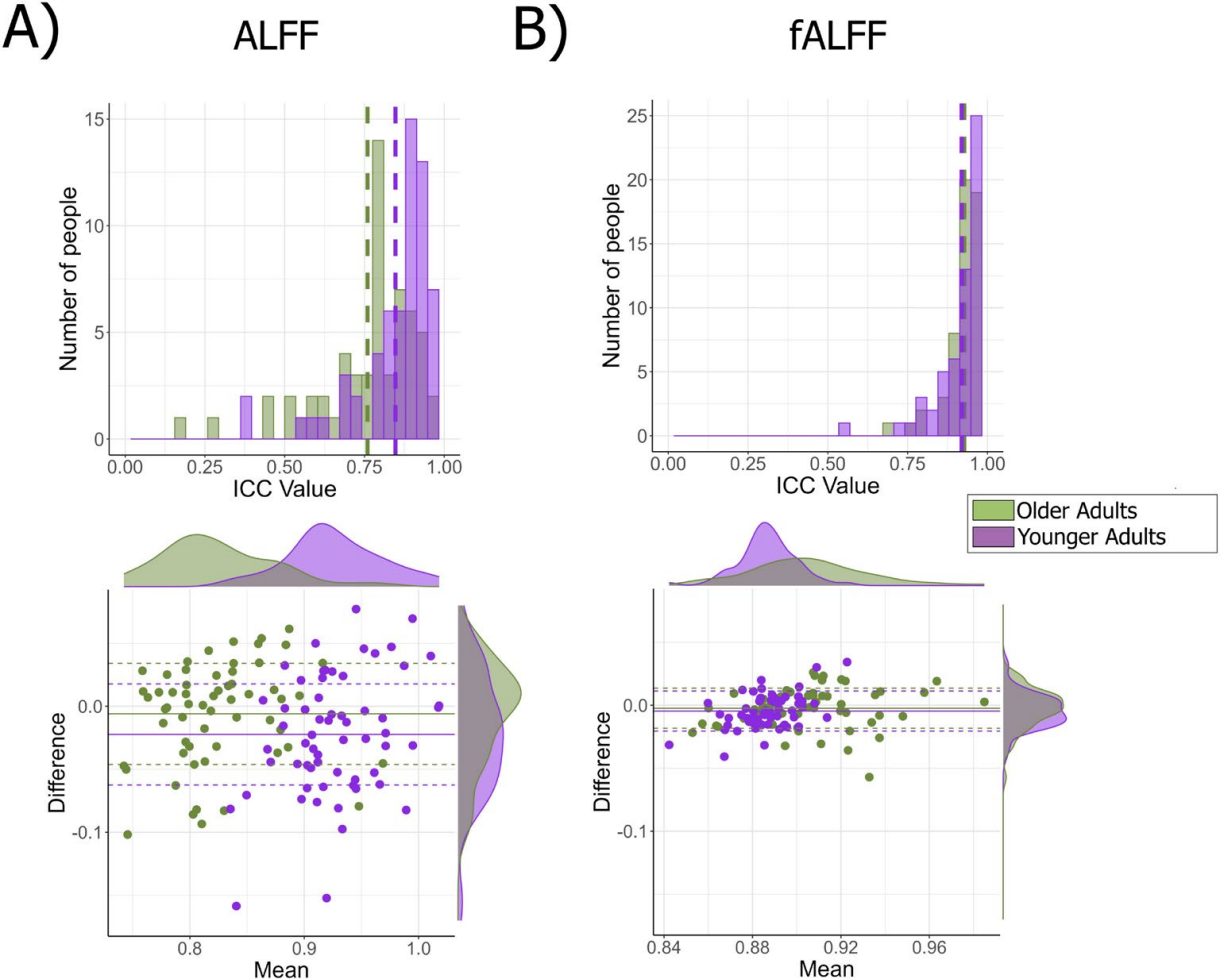
Reproducibility assessment of ALFF and fALFF. Assessment of cerebellar ROI reproducibility using Intraclass Correlation Coefficients (ICCs) for ALFF (left column) and fALFF (right column) in the cerebellum for OA and YA. ICC values and Bland–Altman plots are reflective of repeatability over the entire cerebellum for each participant. (A) Cerebellar ICC and average inter‐session Bland–Altman for ALFF. (B) Cerebellar ICC and average inter‐session Bland–Altman for fALFF.

Bland–Altman plots of average cerebellar ALFF showed clear group‐level differences (OA *N* = 59, μALFF = 0.824; YA *N* = 61, μALFF = 0.927), with YA demonstrating greater differences across rs‐sessions (OA Δ ALFF = −0.022, YA Δ ALFF = −0.006) (Figure 4A). Forty‐eight percent of OA and 79% of YA had an ICC value of 0.8 or greater, indicating moderate to high repeatability for a majority of participants.

Conversely, OA and YA showed similar average cerebellar fALFF across both rs‐sessions (Figure 4B; OA μfALFF = 0.886, YA μfALFF = 0.905) but smaller inter‐session differences (OA Δ fALFF = −0.004, YA Δ fALFF = −0.002). Average cerebellar ICC values increased for both groups, with 93% of participants in both groups demonstrating an ICC of at least 0.8, indicating high repeatability. The increased repeatability of fALFF motivated its use for defining an ROI for FC analysis.

### 3.4 Functional Connectivity Analysis

Using the fALFF Crus I/II seed as indicated in Figure 3 for FC analysis, regions within the DMN demonstrated significant group‐level differences in functional connectivity after p‐FDR correction across both resting state sessions (Table 1). OA demonstrated greater connectivity to cortical regions compared to YA. These regions were located in the right frontal pole (MNI coordinates: *x* = 61, *y* = −6, *z* = −18), the posterior cingulate gyrus (MNI coordinates: *x* = 52, *y* = −56, *z* = 28), and the right lateral occipital cortex (MNI coordinates: *x* = 23, *y* = 36, *z* = 43). Regions that survived a more lenient threshold of p‐FDR < 0.05 can be found in Table S1. Sex, session, and head movement measured by mFD did not demonstrate a significant correlation with FC.

**TABLE 1:** Multi‐linear mixed‐effects model reporting group‐level differences of FC from the cerebellar seed region, with group as a categorical variable (YA as the reference group) and sex, mFD, and session as covariates for the selected cohort after controlling for random effects (*N* = 120; YA = 61, OA = 59 after quality control).

Effect	β	±SE	*T*	*p*	p‐FDR corrected
Right frontal pole (MNI: *x* = 61, *y* = −6, *z* = −18) Network 16					
Group^a^	1.167	0.219	−5.327	0.0000	0.0000
mFD	−4.438	2.008	−2.210	0.0280	0.1620
Sex^b^	0.423	0.193	2.188	0.0297	0.2318
Session	0.018	0.138	0.129	0.8972	0.9771
Right lateral occipital cortex (MNI: *x* = 23, *y* = 36, *z* = 43) Network 16					
Group^a^	1.104	0.229	−4.814	0.0000	0.0001
mFD	−3.569	2.104	−1.696	0.0912	0.3282
Sex^b^	0.167	0.202	0.824	0.4110	0.6678
Session	0.073	0.146	0.503	0.6154	0.9688
Posterior cingulate gyrus (MNI: *x* = 52, *y* = −56, *z* = 28) Network 16					
Group^a^	1.519	0.280	−5.424	0.0000	0.0000
mFD	−2.103	2.573	−0.818	0.4145	0.6890
Sex^b^	0.255	0.247	1.034	0.3024	0.6047
Session	0.151	0.179	0.842	0.4005	0.8723

*Note:* In this model, FC between the Crus II cerebellar seed and the listed ROIs is the dependent variable. Only ROIs that pass p‐FDR < 0.001 for the group effect are shown. The independent variables are listed below.

Abbreviations: mFD, mean framewise displacement; p‐FDR, positive false discovery rate; SE, standardized error; β, standardized beta coefficient.

^a^ The difference in the reported ROI between age groups, with Young Adults as the reference group.

^b^ The difference in the reported ROI between sex, with male participants as the reference group.

## 4 Discussion

This study evaluated ALFF and fALFF as potential biomarkers of healthy aging in the cerebellum. ALFF demonstrated significant group‐level differences in Lobules I–V, IX, and X (Figure 3) along with a moderate effect size, suggesting fair sensitivity (Sullivan and Feinn 2012) to aging differences. However, these measures were less reproducible when compared to fALFF metrics, as shown in ICC calculations and Bland–Altman plots (Figure 4). fALFF was robust and was greater bilaterally in Crus I and II within the cerebellum in older populations (Figure 3) as a potential spatial signature of typical aging. The largest significant cluster of group‐level fALFF differences in right Crus I/II revealed that OA generally has lower FC to this region, particularly within the Default Mode Network (DMN).

### 4.1 Physiological Interpretation of ALFF and fALFF

ALFF and fALFF quantify the BOLD signal and reflect both neuronal and cerebrovascular contributions. ALFF is the absolute amplitude of low‐frequency BOLD fluctuations (0.008–0.1 Hz) and is therefore sensitive to vascular and venous influences, including cardiac and respiratory effects (Birn et al. 2006; Chang et al. 2009; Esteves et al. 2025; Tong et al. 2012), pulsatility (Glover et al. 2000; Kassinopoulos and Mitsis 2021), and proximity to draining veins (Di, Kannurpatti, et al. 2013; Zuo et al. 2010). In contrast, fALFF expresses low‐frequency power as a ratio to the entire frequency spectrum, reducing the effect of systemic noise (Egorova et al. 2017; Huck et al. 2023; Zuo et al. 2010). It should be noted that venous influences on the BOLD signal are not restricted to one frequency band. They affect the whole spectrum, meaning normalization does not fully eliminate their impact. However, studies investigating venous bias on different BOLD metrics have found that this effect is significantly reduced in fALFF compared to ALFF (Huck et al. 2023). This is especially relevant for the cerebellum, which contains numerous draining veins within its compact structure (de Miquel 2021).

BOLD sign and magnitude are affected by cerebrovascular factors such as cerebral blood flow (CBF) (Shin et al. 2007) and cerebrovascular reactivity (CVR) (De Vis et al. 2018; Gao et al. 2025), resulting in corresponding changes in (f)ALFF magnitude. Notably, these cerebrovascular measures are affected by age, where CBF and CVR decrease with greater age (Tsvetanov, Henson, and Rowe 2021). Studies that have investigated BOLD amplitude (specifically, resting state functional amplitude; RSFA, a measure of BOLD amplitude that is proportional to ALFF (Chen and Gauthier 2021; Kannurpatti and Biswal 2008)) have attributed age‐related RSFA differences to physiology as opposed to neuronal influence (Garrett et al. 2017; Henson et al. 2024; Tsvetanov et al. 2015). A study by Tsvetanov and colleagues (Tsvetanov, Henson, Jones, et al. 2021) found that measures of cardiovascular health and CBF were sufficient to account for variance in RSFA across age. Atrophy was not sufficient in explaining age‐related differences in RSFA, suggesting limited neuronal contributions to the BOLD amplitude. In the context of understanding group‐level differences, this information suggests that while ALFF may contain neuronal information, it is likely to be dominated by physiology. On the other hand, while fALFF is more likely to reflect neuronally relevant information, it does not completely remove cerebrovascular or venous effects.

### 4.2 Interpretation and Reliability of ALFF Results

Based on our findings and existing work regarding aging and BOLD amplitude, we believe that cerebellar ALFF primarily reflects vascular differences between age groups. YA showed greater ALFF in medial cerebellar regions, which are proximal to the fourth ventricle. Signal measured in these regions of the cerebellum may be particularly susceptible to acquisition‐related effects, including T2* decay and spatial blurring along the phase‐encoding direction, resulting in greater apparent BOLD signal amplitude and reduced spatial specificity. Consistent with this interpretation, clusters that showed group‐level ALFF differences (YA > OA) had a large overlap with regions that showed a gBOLD correlation in the same direction. These regions had a strong visual correspondence with regions of high venous probability (Huck et al. 2019), suggesting confounds with vascular signal. Moreover, OA demonstrated greater tSNR group‐level differences than YA. This suggests that lower ALFF in OA is unlikely to reflect reduced data quality and instead may reflect reduced low‐frequency neural variability with age, or increased vascular stiffness resulting in more stable signal baseline and lower ALFF.

The latter interpretation is consistent with prior work that has found ALFF to be more sensitive specifically to venous bias than fALFF (Huck et al. 2023). The contributions of physiology to ALFF serve as a potential explanation as to why test–retest reliability was lower for both groups, as reflected in ICC values and Bland–Altman plots (Figure 3). However, the effect size of ALFF differences (*d* = 0.54) indicates a meaningful difference in physiology between YA and OA. Controlling for GMVp did not change the spatial extent of age‐related ALFF clusters, suggesting that BOLD amplitude differences are not influenced by structural differences. Although ALFF showed spatial similarity to gBOLD correlations, gBOLD did not significantly correlate with ALFF; therefore, there is no strong bias from scanner drift, head movement, or physiological noise. Measures of CVR and CBF influence the amplitude of the BOLD signal and would help to disentangle this interpretation. Moreover, these factors are influenced by aging but were not included in this study due to available scan limitations. Future work investigating these physiological metrics and ALFF in the cerebellum can more directly confirm the extent to which ALFF patterns reflect vascular or neuronal aging.

### 4.3 Interpretation and Reliability of fALFF Results

We identified greater lateralized fALFF in OA within Crus I and II regions (Figure 3), regions that have demonstrated the greatest functional correlation/structural connection between prefrontal/frontal regions (Alasmar et al. 2025; Bernard et al. 2012; Buckner et al. 2011; Krienen and Buckner 2009; O'Reilly et al. 2010). Given that aging is associated with disrupted DMN integrity and reduced spontaneous cortical activity (Sambataro et al. 2010; Schroeter et al. 2004; Vidal‐Piñeiro et al. 2014; Xing 2021; Zhong and Chen 2022), we anticipated reduced fALFF in cerebellar DMN regions. Instead, we observed higher fALFF in OA. It is important to note that higher fALFF values reflect differences in the relative contribution of low‐frequency power rather than a direct measure of neuronal activity alone. A decrease in high‐frequency BOLD signal amplitude can also lead to lower ALFF values while simultaneously elevating fALFF. fALFF values demonstrated high test–retest reliability (Figure 4) and cluster extents of group differences were not affected after taking GMVp or gBOLD into account (Figure 2). Regions that demonstrated fALFF group‐level differences did not overlap with regions that showed tSNR differences; however, OA overall demonstrated greater tSNR across the cerebellum. Higher tSNR may reflect reduced high‐frequency noise, increasing the relative contribution of low‐frequency power and resulting in higher fALFF values.

### 4.4 Low Frequency Amplitude and Functional Connectivity

Our findings demonstrate elevated cerebellar BOLD signal in OA and increased connectivity between fALFF‐defined cerebellar regions and subregions within the DMN. Previous studies have noted decreased DMN connectivity with age (Damoiseaux 2017; Jones et al. 2011; Park and Reuter‐Lorenz 2009; Varangis et al. 2019). However, fALFF is typically interpreted to reflect increased spontaneous neural activity partly influenced by functional connectivity (Zou et al. 2008). Age‐related changes in FC are not uniform across all regions or networks, and have been reported to vary within the DMN itself with aging (Campbell et al. 2013; Huang et al. 2015). Moreover, FC differences with age are heterogeneous, with longitudinal studies reporting non‐linear relationships between FC and age (Luo et al. 2019; Malagurski et al. 2022) as well as cerebellar structure and age (Romero et al. 2021). FC analysis of cortico‐cerebellar networks by Bernard and colleagues (Bernard et al. 2013) reported that older adults were more likely to demonstrate increased connectivity to frontal regions, a pattern interpreted as reflecting altered or increased engagement of cerebellar networks. Our observed increase in FC associated with fALFF‐defined regions may reflect similar alterations in cortico‐cerebellar connectivity across age groups.

There are several non‐neuronal explanations as to why we see increased rather than decreased FC in OA. Reduced BOLD signal variability in OA (as indicated by increased tSNR and decreased ALFF), greater head motion (with Crus II demonstrating increased sensitivity to slow head motion (Tomasi and Volkow 2012)), or differences in vascular compliance may all result in spurious long‐range connectivity. Notably, while both groups demonstrated minimal head motion, both OA and YA had a negative correlation between mFD and FC. Van Dijk et al. (2012) found that head motion resulted in varied functional connectivity changes depending on the network of interest, with DMN connectivity decreasing with increasing head motion. Together, these findings suggest that increased cerebellar FC in older populations may arise from a complex interaction between age‐related network reorganization and non‐neuronal age‐related factors that influence BOLD variability and amplitude.

### 4.5 Limitations

There are several limitations to our study. First, image acquisition site and protocol are confounded with the separate age cohorts. YA were scanned at a single site with one protocol, while OA were scanned across multiple sites with a different protocol. The HCP site collection and data acquisition were developed to minimize differences between the separate datasets (Harms et al. 2018) but there remain inherent differences in signal due to protocol and hardware (Adhikari et al. 2019) that may be confounded with the separate cohorts. tSNR has been used to compare differences in HCP data quality attributes (Chan et al. 2026) and was used in our study to at least partially provide insight into image quality differences. Our finding of greater tSNR in OA aligns with other studies that have compared image quality between the HCP cohorts (Chan et al. 2026), however, we cannot conclude if these differences are cohort‐ or acquisition‐driven. Future studies with harmonized protocol would help to confirm our results.

Additionally, our sample size of *N* = 120 is modest compared to larger fMRI studies. While this limits generalizability and statistical power, the effect size and our reliability analysis suggest that these results are consistent and repeatable. As mentioned earlier, existing literature has reported differences within DMN connectivity with age (Sambataro et al. 2010; Schroeter et al. 2004; Vidal‐Piñeiro et al. 2014; Xing 2021; Zhong and Chen 2022), but we did not find group‐level FC differences in our analysis. Other cortico‐cerebellar FC studies have found connectivity differences with age (Bernard et al. 2013; de Uwisengeyimana et al. 2020). This may reflect methodological differences, as these studies used whole cerebellar anatomical lobules as seed regions and used voxel‐wise comparison, while we used a data‐driven approach to define a seed and conduct seed‐to‐ROI analysis. It is also important to note that using group differences in fALFF may bias our FC analysis to reflect group differences. While prior work has utilized similar methods (Di, Kim, et al. 2013; Fan et al. 2019; Wang et al. 2021), future work should employ independent datasets or cross‐validation approaches to confirm our results. Additionally, to provide a physiological interpretation of cerebellar (f)ALFF, we used gBOLD as an indirect measure of physiology. Direct physiological measures such as breathing and heart rate recordings can be used, but these can be corrupted and difficult to use for removal of physiological confounds (Addeh et al. 2023; Addeh, Vega, et al. 2025). gBOLD has been used as a general measure of physiology (Liu et al. 2017). We did not regress this signal out using global signal regression as it can contain information important to aging effects, but included it as a covariate in our analysis. Future studies with a larger cohort, direct measurements of physiology, and complementary FC approaches will be important in confirming the findings presented here.

## 5 Conclusions

Our study provides a potential biomarker of healthy aging in the cerebellum using ALFF as a measure of vascular differences and fALFF as a potential measure of neuronal cerebellar differences. In the context of aging, there are several hypotheses as to how circuitry within the brain varies; however, these hypotheses are limited to cortical regions (Cabeza 2002; Davis et al. 2008; Reuter‐Lorenz et al. 1999; Reuter‐Lorenz and Cappell 2008) and do not include subcortical regions (MacDonald and Pike 2021). In summary, we found that increased BOLD fluctuations in subregions of the cerebellum with age coincide with an increased relative FC to the cortex. Our findings highlight the need to include the cerebellum, among other subcortical structures, in functional models of compensatory or dedifferentiation changes in aging.

## Funding

This work was supported by the National Science Foundation (2036201), the National Institutes of Health (P30 AG 072972), the Natural Sciences and Engineering Research Council of Canada (RGPIN‐2020‐06812, DGECR‐2020‐00146), the Heart and Stroke Foundation of Canada (HNC 170723), and the Fonds de Recherche du Québec—Santé (FRQS CB Junior 2 249443).

## Consent

My article reports human subjects. Recruitment meets scientific requirements & HBMs expectation of inclusivity. This study involved research with human participants. Inclusion and exclusion criteria were scientifically justified and included factors such as age (18–35 vs. 60–90), neurological status, and existing MRI availability.

## Conflicts of Interest

The authors declare no conflicts of interest.

## Supporting information


**Figure S1:** The Venous Probability Atlas (VENAT, left; (Huck et al. 2019)) projected on SUIT space (right).
**Table S1:** Multi‐linear mixed‐effects model reporting group‐level differences of FC from the cerebellar seed region, with group as a categorical variable and sex, mFD, and session as covariates for the selected cohort after controlling for random effects (*N* = 120; YA = 61, OA = 59 after quality control). In this model, FC between the Crus II cerebellar seed and the listed ROIs are the dependent variables. Only ROIs that pass p‐FDR < 0.05 for the group effect are shown. The independent variables are listed below.

## Data Availability

The data that support the findings of this study are available from the corresponding author upon reasonable request.
