# Supplementary material for: Age‐Related Low Frequency Amplitude Differences in Resting‐State Blood Oxygenation Level‐Dependent Signal in the Cerebellum
Source: Hum Brain Mapp. 2026 May 10;47(7):e70541. doi: 10.1002/hbm.70541 (PMC13158378; doi:10.1002/hbm.70541)
Supplement: Supplementary file 1 — Figure S1: The Venous Probability Atlas (VENAT, left; (Huck et al. 2019)) projected on SUIT space (right). Table S1: Multi‐linear mixed‐effects model reporting group‐level differences of FC from the cerebellar seed region, with group as a categorical variable and sex, mFD, and session as covariates for the selected cohort after controlling for random effects (N = 120; YA = 61, OA = 59 after quality control). In this model, FC between the Crus II cerebellar seed and the listed ROIs are the dependent variables. Only ROIs that pass p‐FDR < 0.05 for the group effect are shown. The independent variables are listed below. [file HBM-47-e70541-s001.docx]

**Supplementary**


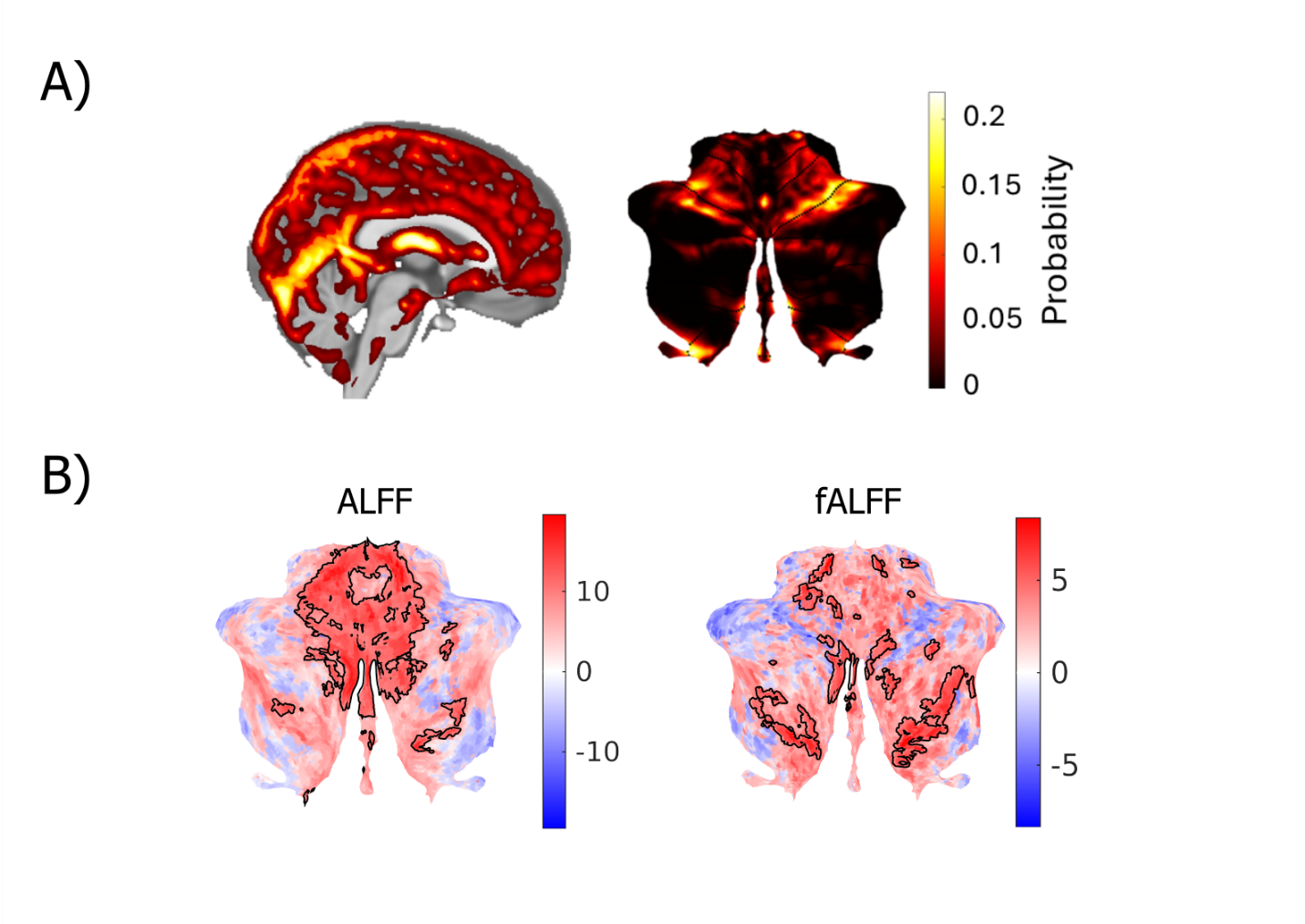


**Supplementary Figure 1.** The Venous Probability Atlas (VENAT, left; (Huck et al., 2019)) projected on SUIT space (right).

**Supplementary Table S1**. Multi-linear mixed-effects model reporting group-level differences of FC from the cerebellar seed region, with group as a categorical variable and sex, mFD, and session as covariates for the selected cohort after controlling for random effects (N=120; YA = 61, OA = 59 after quality control). In this model, FC between the Crus II cerebellar seed and the listed ROIs are the dependent variables. Only ROIs that pass p-FDR<0.05 for the group effect are shown. The independent variables are listed below.

|  |  | | | | |  |
| --- | --- | --- | --- | --- | --- | --- |
| **Effect** | | **β** | **± SE** | ***T*** | ***p*-value** | ***p-FDR corrected*** |
|  | | ***Cerebellum Left VIIIb (MNI: x= -22, y= -48, z= -56)***  ***Network 3*** | | | | |
| Group^a^ | | -0.395 | 0.129 | 3.064 | 0.0024 | 0.0201 |
| mFD | | 0.350 | 1.205 | 0.290 | 0.7718 | 0.8898 |
| Sex | | 0.121 | 0.113 | 1.072 | 0.2848 | 0.5905 |
| Session | | 0.012 | 0.093 | 0.129 | 0.8972 | 0.9771 |
|  | | ***Precentral gyrus (MNI: x= -1, y= -23, z= 62)***  ***Network 3*** | | | | |
| Group^a^ | | 0.473 | 0.158 | -2.989 | 0.0031 | 0.0201 |
| mFD | | -6.060 | 1.473 | -4.113 | 0.0001 | 0.0049** |
| Sex | | 0.154 | 0.139 | 1.108 | 0.2689 | 0.5905 |
| Session | | -0.205 | 0.110 | -1.869 | 0.0629 | 0.5943 |
|  | | ***Lateral occipital cortex (MNI: x= -34, y= -64, z=18)***  ***Network 5*** | | | | |
| Group^a^ | | -0.527 | 0.164 | 3.209 | 0.0015 | 0.0185 |
| mFD | | 0.718 | 1.538 | 0.467 | 0.6412 | 0.8232 |
| Sex | | 0.012 | 0.144 | 0.086 | 0.9318 | 0.9770 |
| Session | | -0.025 | 0.120 | -0.210 | 0.8340 | 0.9771 |
|  | | ***Cerebellum Left IX (MNI: x= -7, y= -55, z= -49)***  ***Network 7*** | | | | |
| Group^a^ | | -0.304 | 0.101 | 3.016 | 0.0028 | 0.0201 |
| mFD | | -0.698 | 0.929 | -0.751 | 0.4533 | 0.7127 |
| Sex | | -0.069 | 0.089 | -0.773 | 0.4403 | 0.6739 |
| Session | | 0.093 | 0.066 | 1.419 | 0.1572 | 0.6541 |
|  | | ***Cerebellum Right Crus I (MNI: x= 42, y= -49, z= -32)***  ***Network 8*** | | | | |
| Group^a^ | | -0.451 | 0.155 | 2.914 | 0.0039 | 0.0234 |
| mFD | | 0.576 | 1.405 | 0.410 | 0.6825 | 0.8460 |
| Sex | | -0.293 | 0.137 | -2.139 | 0.0334 | 0.2318 |
| Session | | 0.143 | 0.093 | 1.548 | 0.1230 | 0.6499 |
|  | | ***Cerebellum Right X (MNI: x= -40, y= -52, z= -35)***  ***Network 8*** | | | | |
| Group^a^ | | -0.446 | 0.149 | 2.990 | 0.0031 | 0.0201 |
| mFD | | 0.166 | 1.341 | 0.124 | 0.9017 | 0.9407 |
| Sex | | -0.352 | 0.132 | -2.662 | 0.0083 | 0.1437 |
| Session | | 0.112 | 0.085 | 1.317 | 0.1890 | 0.6686 |
|  | | ***Left middle frontal gyrus (MNI: x= -41, y= 24, z= 40) Network 13*** | | | | |
| Group^a^ | | 1.036 | 0.357 | -2.903 | 0.0040 | 0.0234 |
| mFD | | -5.107 | 3.075 | -1.661 | 0.0981 | 0.3401 |
| Sex | | 0.519 | 0.320 | 1.619 | 0.1068 | 0.3612 |
| Session | | -0.029 | 0.169 | -0.172 | 0.8633 | 0.9771 |
|  | | ***Cerebellum Left Crus II (MNI: x= 18, y= -38, z= -45)***  ***Network 15*** | | | | |
| Group^a^ | | -0.365 | 0.119 | 3.055 | 0.0025 | 0.0201 |
| mFD | | -0.909 | 1.119 | -0.812 | 0.4174 | 0.6890 |
| Sex | | 0.015 | 0.105 | 0.144 | 0.8857 | 0.9595 |
| Session | | 0.098 | 0.088 | 1.120 | 0.2639 | 0.8171 |
|  | | ***Anterior middle temporal gyrus (MNI: x= -13, y= -85, z= -40)***  ***Network 16*** | | | | |
| Group^a^ | | 0.820 | 0.260 | -3.154 | 0.0018 | 0.0189 |
| mFD | | 1.733 | 2.337 | 0.741 | 0.4592 | 0.7127 |
| Sex | | -0.134 | 0.231 | -0.582 | 0.5609 | 0.7479 |
| Session | | 0.113 | 0.147 | 0.770 | 0.4423 | 0.9008 |
|  | | ***Right frontal pole (MNI: x= 61, y= -6, z= -18)***  ***Network 16*** | | | | |
| Group^a^ | | 1.167 | 0.219 | -5.327 | 0.0000 | 0.0000 |
| mFD | | -4.438 | 2.008 | -2.210 | 0.0280 | 0.1620 |
| Sex | | 0.423 | 0.193 | 2.188 | 0.0297 | 0.2318 |
| Session | | 0.018 | 0.138 | 0.129 | 0.8972 | 0.9771 |
|  | | ***Right lateral occipital cortex (MNI: x= 23, y= 36, z= 43)***  ***Network 16*** | | | | |
| Group^a^ | | 1.104 | 0.229 | -4.814 | 0.0000 | 0.0001 |
| mFD | | -3.569 | 2.104 | -1.696 | 0.0912 | 0.3282 |
| Sex | | 0.167 | 0.202 | 0.824 | 0.4110 | 0.6678 |
| Session | | 0.073 | 0.146 | 0.503 | 0.6154 | 0.9688 |
|  | | ***Left superior frontal gyrus (MNI: x= -21, y= 29, z = 45) Network 16*** | | | | |
| Group^a^ | | 0.665 | 0.254 | -2.622 | 0.0093 | 0.0484 |
| mFD | | -7.029 | 2.292 | -3.066 | 0.0024 | 0.0504 |
| Sex | | 0.590 | 0.225 | 2.625 | 0.0092 | 0.1437 |
| Session | | 0.110 | 0.147 | 0.746 | 0.4566 | 0.9008 |
|  | | ***Right angular gyrus (MNI: x= -44, y= -68, z= 37)***  ***Network 16*** | | | | |
| Group^a^ | | 0.993 | 0.350 | -2.838 | 0.0049 | 0.0270 |
| mFD | | -7.930 | 3.124 | -2.538 | 0.0118 | 0.1022 |
| Sex | | 0.522 | 0.311 | 1.676 | 0.0951 | 0.3499 |
| Session | | 0.045 | 0.191 | 0.235 | 0.8143 | 0.9771 |
|  | | ***Posterior cingulate gyrus (MNI: x= 52, y= -56, z= 28)***  ***Network 16*** | | | | |
| Group^a^ | | 1.519 | 0.280 | -5.424 | <0.0001 | <0.0001 |
| mFD | | -2.103 | 2.573 | -0.818 | 0.4145 | 0.6890 |
| Sex | | 0.255 | 0.247 | 1.034 | 0.3024 | 0.6047 |
| Session | | 0.151 | 0.179 | 0.842 | 0.4005 | 0.8723 |
|  | | ***Medial superior frontal gyrus (MNI: x= 0, y= 50, z= 30)***  ***Network 16*** | | | | |
| Group^a^ | | 0.737 | 0.216 | -3.405 | 0.0008 | 0.0115 |
| mFD | | -2.731 | 1.986 | -1.375 | 0.1704 | 0.4192 |
| Sex | | 0.255 | 0.191 | 1.336 | 0.1828 | 0.4648 |
| Session | | 0.009 | 0.137 | 0.064 | 0.9492 | 0.9771 |
|  | | ***Paracingulate gyrus (MNI: x= 0, y= 50 z= 5)***  ***Network 16*** | | | | |
| Group^a^ | | 0.713 | 0.200 | -3.561 | 0.0004 | 0.0077 |
| mFD | | -5.279 | 1.845 | -2.862 | 0.0046 | 0.0597 |
| Sex | | 0.175 | 0.177 | 0.988 | 0.3240 | 0.6115 |
| Session | | -0.062 | 0.130 | -0.481 | 0.6312 | 0.9688 |
|  | | ***Right posterior middle temporal gyrus (MNI: x= 61, y= -25, z= -6)***  ***Network 17*** | | | | |
| Group^a^ | | 0.679 | 0.222 | -3.057 | 0.0025 | 0.0201 |
| mFD | | 1.315 | 2.081 | 0.632 | 0.5280 | 0.7845 |
| Sex | | 0.391 | 0.195 | 2.004 | 0.0462 | 0.2529 |
| Session | | 0.099 | 0.163 | 0.612 | 0.5414 | 0.9008 |
|  | | ***Left angular gyrus (MNI: x= -52, y= -54, z= 28)***  ***Network 17*** | | | | |
| Group^a^ | | 1.185 | 0.371 | -3.193 | 0.0016 | 0.0185 |
| mFD | | -8.282 | 3.158 | -2.623 | 0.0093 | 0.0878 |
| Sex | | 0.626 | 0.334 | 1.874 | 0.0622 | 0.2941 |
| Session | | -0.162 | 0.168 | -0.964 | 0.3361 | 0.8544 |
|  | | ***Right anterior middle temporal gyrus (MNI: x= 51, y= 4, z= -30)***  ***Network 17*** | | | | |
| Group^a^ | | 0.677 | 0.173 | -3.903 | 0.0001 | 0.0028 |
| mFD | | -3.576 | 1.598 | -2.238 | 0.0262 | 0.1600 |
| Sex | | 0.361 | 0.153 | 2.358 | 0.0192 | 0.1997 |
| Session | | -0.031 | 0.113 | -0.271 | 0.7867 | 0.9771 |
|  | | ***Left posterior middle temporal gyrus (MNI: x= -56, y= -11, z= -19)***  ***Network 17*** | | | | |
| Group^a^ | | 0.748 | 0.193 | -3.878 | 0.0001 | 0.0028 |
| mFD | | -4.327 | 1.737 | -2.491 | 0.0134 | 0.1073 |
| Sex | | 0.545 | 0.171 | 3.185 | 0.0016 | 0.1437 |
| Session | | -0.066 | 0.110 | -0.595 | 0.5524 | 0.9008 |

*mFD: mean framewise displacement; β: standardized beta coefficient; SE: standardized error; p-FDR: positive false discovery rate*

*^a^The difference in the reported ROI between groups, with Young Adults as the reference group.*

*** Indicates p<0.01 for a non-Group effect.*
